# Standardization of Fecal Metabolomics Using Microbiome Preservation Kits: Implications for Multiomics Integration

**DOI:** 10.1155/ianc/8551545

**Published:** 2025-12-17

**Authors:** Yu Ra Lee, Jae-Ho Park, Hye Hyun Yoo, Inwook Choi, Ho-Young Park

**Affiliations:** ^1^ Food Functionality Research Division, Korea Food Research Institute, Wanju-Gun, 55365, Jeollabuk-do, Republic of Korea, kfri.re.kr; ^2^ Pharmacomicrobiomics Research Center, College of Pharmacy, Hanyang University, Ansan, 15588, Republic of Korea, hanyang.ac.kr; ^3^ Department of Food Biotechnology, Korea National University of Science and Technology, Daejeon, 34113, Republic of Korea

**Keywords:** dry weight, feces, metabolite, normalization, wet weight

## Abstract

With the advancement of multiomics technologies and cohort study designs, integrative omics research is increasingly applied to human health and nutrition. However, optimal storage and preprocessing of labile biological samples, particularly feces, remain challenging. In this study, we systematically evaluated three normalization methods—wet weight, dry weight, and protein quantification—for quantitative metabolomic profiling of fecal samples, using 41 metabolites. Fresh fecal samples from three healthy individuals showed high reproducibility, with 24 metabolites exhibiting a coefficient of variation (CV) below 30 for both wet and dry weight normalization. Fecal samples from 20 obese patients collected using the OMNIgene·GUT kit demonstrated improved reproducibility with wet weight normalization (20 metabolites, CV < 30) and protein quantification normalization (19 metabolites, CV < 30), whereas dry weight normalization yielded no metabolites meeting the CV < 30 criterion. Direct analysis of the kit solution without a drying step further enhanced chromatographic clarity, highlighting practical considerations for large‐scale studies. Overall, wet weight normalization consistently minimized variation across sample types, providing a robust and standardized framework for fecal metabolite profiling. These findings demonstrate that the OMNIgene·GUT kit is compatible with broad‐spectrum metabolomic analyses and support its integration into multiomics workflows. By establishing reproducible normalization protocols, this study provides the foundation for accurate, comparable, and scalable fecal metabolomics in both clinical and nutritional research settings.

## 1. Introduction

Multiomics analyses, integrating metagenomics and metabolomics, have emerged as powerful tools in advancing precision medicine and personalized nutrition. Gut microbiota are deeply involved in human health, influencing immune regulation, neural signaling, and energy metabolism, and their composition is highly responsive to dietary and lifestyle factors [1–4]. Integrative approaches that combine microbial and metabolite profiling enable a deeper understanding of microbe–metabolite–host interactions and their implications for human health and disease [5, 6]. Similarly, integrated multiomics approaches have been applied to elucidate relationships between microbial communities, metabolic status, and host phenotypes in other systems, such as livestock nutrition [7]. Moreover, analyzing metabolites produced by the intestinal microbiota is essential from a physiological perspective [8].

Feces represent a complex biological matrix that reflects the interplay between the gut microbiota and the host, providing valuable insights into microbial activity, nutrient metabolism, and host phenotypes. However, fecal metabolite analysis is highly sensitive to sample collection, transportation, storage, and extraction conditions [9–12]. Although several studies have optimized storage methods and extraction protocols [13–15], no standardized protocol exists for the quantitative normalization of fecal metabolites. Recently, the OMNIgene·GUT kit has gained popularity in large‐scale cohort studies due to its convenience and ability to stabilize microbial DNA, enabling downstream microbiome analyses such as 16S rRNA sequencing and targeted metabolite profiling, particularly for short‐chain fatty acids [16, 17]. Nevertheless, limited research has evaluated the kit’s applicability to broad‐spectrum metabolomics, and variability in sample mass inherent to self‐collection presents a challenge for achieving reproducible quantitative results.

In this study, we systematically evaluated normalization strategies for quantitative metabolomic profiling of fecal samples, comparing fresh stool with samples collected using the OMNIgene·GUT kit. To our knowledge, this is the first study to assess reproducibility and standardization of normalization methods with the OMNIgene·GUT kit for broad‐spectrum metabolite profiling. Our findings provide practical guidance for establishing standardized workflows for fecal metabolite analyses, supporting reproducible and accurate results in future microbiome and metabolome studies.

## 2. Materials and Methods

### 2.1. Chemicals

Reference standards were obtained from Sigma‐Aldrich (St. Louis, MO, USA), Tokyo Chemical Industry (Tokyo, Japan), and Cayman Chemical (Ann Arbor, MI, USA). LC–MS‐grade acetonitrile (ACN), methanol (MeOH), and water were obtained from Fisher Scientific (Waltham, MA, USA).

### 2.2. Fecal Sample Collection

This study was approved by the Institutional Review Board (IRB) of Yonsei Medical School, Severance Hospital (IRB‐No. 4‐2022‐0645), Seoul, Korea. For the first fecal sample analysis, fecal samples (approximately 50 mg) were obtained from normal controls (*n* = 3) in a 1.5 mL tube. Samples were stored at −80°C before analysis. For the second fecal sample analysis, samples were obtained from 20 patients categorized as obese using the OMNIgene·GUT kit (DNA Genotek, Ottawa, Canada) and immediately frozen at −80°C. To perform microbiome and metabolite analyses using the same sample, feces were placed in the OMNIgene·GUT kit, and fecal metabolite analysis was performed using the kit solution. Fecal samples were collected in an OMNIgene·GUT tube using the supplied applicator according to the manufacturer’s instructions.

### 2.3. Sample Preparation Using Fresh Feces

Fresh fecal samples were collected in tubes, and their weights were measured before transferring them to new tubes. Approximately 50 mg of the fecal sample was used for the experiment. In each tube, 20 μL of IS and 980 μL of 50% MeOH were added. After extraction, the samples were sonicated three times for 10 min in an ice bath. Thereafter, the samples were centrifuged at 18,000 ×*g* for 10 min. After centrifugation, the supernatant was transferred (750 μL) and pooled in a 10 mL tube. The pooled samples (750 μL) were divided a total of 9 times prior to normalization.

For the wet weight normalization and protein quantification normalization tests, each supernatant sample was weighed (750 μL) and filtered using a PTFE filter (0.2 μm, 13 mm diameter; Whatman, USA). Protein quantification was performed using a DC protein assay (Bio‐Rad, RC DC protein assay kit) following the manufacturer’s instructions.

For dry weight normalization, 750 μL of aliquot was dried using N_2_ gas. After, each dried sample was weighed and reconstituted in 750 μL of 50% MeOH. Then, samples were filtered using a PTFE filter. Figure 1(a) outlines the experimental procedure.

Figure 1Schematic outline of sample preparation procedure. (a) Fresh fecal sample. (b) Fecal sample using the OMNIgene·GUT kit.

**Figure:**
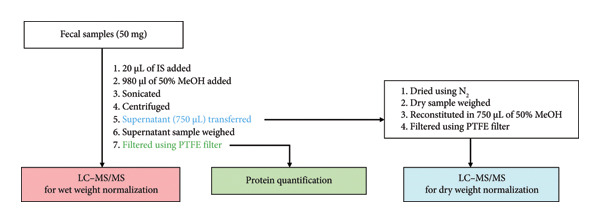
(a)

**Figure:**
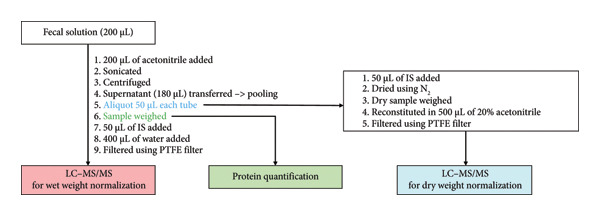
(b)

### 2.4. Sample Preparation Using the OMNIgene·GUT Kit

The solutions in the OMNIgene·GUT kit (200 μL) were added to 200 μL of ACN for extraction. After extraction, the samples were sonicated three times for 10 min in an ice bath. Thereafter, the samples were centrifuged at 18,000 ×*g* for 10 min. After centrifugation, the supernatant (up to 180 μL) was pooled in a 5 mL tube. After, 50 μL of aliquot was taken from each tube for subsequent testing. For the wet weight normalization test, each sample was weighed; 50 μL of IS and 400 μL of water were added; and each sample was filtered using a PTFE filter.

For the protein quantification normalization test, 10 μL of aliquot was added to 10 μL of water for dilution, and quantification was performed using a DC protein assay (Bio‐Rad, RC DC protein assay kit), following the manufacturer’s instructions.

For the dry weight normalization test, 50 μL of aliquot was dried using N_2_ gas. After, each dried sample was weighed; 50 μL of IS, 50 μL of ACN, and 400 μL of water were added; and samples were filtered using a PTFE filter. Figure 1(b) outlines the overall experimental procedure.

### 2.5. Instrumental and Chromatographic Conditions

The LC–MS/MS analysis was performed using an ACQUITY H‐Class UPLC system (Waters) coupled to a Xevo TQ MS instrument (Waters). Chromatographic separation was performed using an ACQUITY UPLC BEH C18 column (2.1 × 100 mm, 1.7 μm) at 350 μL/min. A gradient eluent (A: 0.1% formic acid in water; B: 0.1% formic acid in ACN) was used. The gradient elution system was controlled as follows: 0–17.5 min, linear increase from 5% to 95% B; 17.5–19 min, 95%–89% B; 19–19.5 min, linear decrease from 89% to 5% B; and 19.5–22 min, 5% B. The column and autosampler temperatures were maintained at 40°C and 8°C, respectively. In Table S1, multiple reaction monitoring conditions used in the analysis were shown.

## 3. Results and Discussion

**Figure 2:**
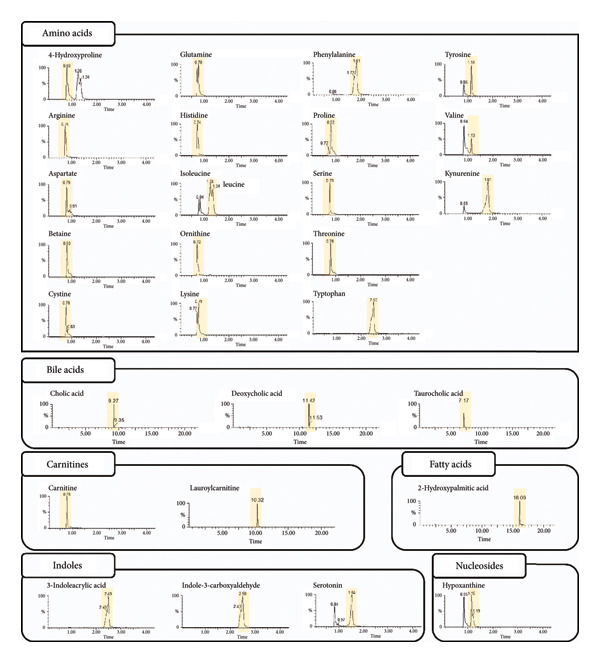
Chromatograms of the analyte obtained using LC–MS/MS.

We systematically evaluated three normalization methods—wet weight, dry weight, and protein quantification—for quantitative metabolomic profiling of fecal samples, using both fresh stool from healthy individuals and samples collected with the OMNIgene·GUT kit (Tables 1, 2, 3, 4). To minimize technical variability during preprocessing, all samples were pooled after extraction. A total of 41 metabolites were analyzed, including amino acids, bile acids, carnitines, indoles, nucleosides, a carboxylic acid, and a steroid. Chromatograms were acquired in multiple monitoring mode (Figure 2), and peak quality, baseline stability, and reproducibility were carefully assessed.

**Table 1:** Amino acids.

Normalization types	Wet weight		Dry weight		Protein quantification	
Metabolites	Fresh feces	OMNIgene·GUT kit	Fresh feces	OMNIgene·GUT kit	Fresh feces	OMNIgene·GUT kit
Arginine	68.5	**16.28**	115.97	89.83	67.15	**21.43**
Aspartate	35.68	64.26	**17.8**	97.26	**28.39**	50.13
Betaine	124.44	**20.53**	152.76	75.21	80.2	**24.75**
Cystine	150.74	ND	99.07	ND	83.4	ND
Glutamine	**17.16**	**22.44**	**19.59**	93.99	**18.43**	**23.31**
Hippurate	171.29	ND	87.25	ND	171.27	ND
Histidine	**15.46**	50.34	**13.37**	219.21	**15.01**	54.91
Isoleucine	**9.13**	**23.76**	**10.43**	90.52	**7.01**	**22.68**
Leucine	**13.84**	**22.87**	**15.4**	98.9	**5.5**	**22.05**
Lysine	**20.53**	**13.58**	**1.49**	80.51	31.54	**19.02**
*N*‐Acetylglycine	66.68	62.23	34.38	110.94	51.7	60.61
Ornithine	**7.76**	**20.03**	**16.92**	82.57	34.28	**23.04**
Phenylalanine	**11.82**	**11.12**	**14.16**	86.83	**7.41**	**15.39**
Proline	**16.56**	**12.02**	**15.96**	86.49	**2.47**	**8.02**
Serine	**5.88**	37.34	**23.05**	89.76	**10.79**	48.46
Threonine	**8.47**	**25.71**	**14.02**	90.99	**6.06**	**25.82**
*Trans*‐4‐hydroxyproline	**14.82**	**29.38**	**9.66**	101.61	**9.21**	**26.76**
Tryptophan	**13.24**	**16.64**	**15.03**	89.58	**3.76**	**18.63**
Tyrosine	**7.88**	**11.06**	**13.11**	87.34	**15.86**	**16.53**
Valine	**7.53**	**14.47**	**14.48**	87.47	**14.39**	**19.09**
No. of metabolites with CV < 30	**14**	**14**	**15**	0	**13**	**14**

*Note:* Grouped summary of fecal metabolites detected in this study. Metabolites are grouped by chemical classes, and their relative abundance is expressed as a percentage (%). CV values are shown for each metabolite under the three normalization methods. Metabolites with CV values ≤ 30, highlighted in bold, indicate high technical reproducibility.

**Table 2:** Bile acids.

Normalization types	Wet weight		Dry weight		Protein quantification	
Metabolites	Fresh feces	OMNIgene·GUT kit	Fresh feces	OMNIgene·GUT kit	Fresh feces	OMNIgene·GUT kit
Cholic acid	**21.01**	37.45	80.58	102.67	77.33	44.68
Deoxycholic acid	**4.48**	63.33	**18.6**	79.77	**23.51**	75.83
Glycocholic acid	ND	51.33	ND	99.27	ND	60.23
Taurocholic acid	141.89	34.2	115.55	116.94	44.68	32.39
No. of metabolites with CV < 30	2	0	1	0	1	0

*Note:* Grouped summary of fecal metabolites detected in this study. Metabolites are grouped by chemical classes, and their relative abundance is expressed as a percentage (%). CV values are shown for each metabolite under the three normalization methods. Metabolites with CV values ≤ 30, highlighted in bold, indicate high technical reproducibility.

**Table 3:** Carnitines.

Normalization types	Wet weight		Dry weight		Protein quantification	
Metabolites	Fresh feces	OMNIgene·GUT kit	Fresh feces	OMNIgene·GUT kit	Fresh feces	OMNIgene·GUT kit
Carnitine	**13.09**	32.51	**16.29**	113.24	**18.72**	30.61
Butyrylcarnitine	66.55	ND	59.36	ND	72.68	ND
Lauroylcarnitine	**0.56**	**5.76**	**12.57**	87.29	45.98	**6.29**
No. of metabolites with CV < 30	2	1	2	0	1	1

*Note:* Grouped summary of fecal metabolites detected in this study. Metabolites are grouped by chemical classes, and their relative abundance is expressed as a percentage (%). CV values are shown for each metabolite under the three normalization methods. Metabolites with CV values ≤ 30, highlighted in bold, indicate high technical reproducibility.

**Table 4:** Other metabolites.

Normalization types	Wet weight		Dry weight		Protein quantification	
Metabolites	Fresh feces	OMNIgene·GUT kit	Fresh feces	OMNIgene·GUT kit	Fresh feces	OMNIgene·GUT kit
3‐Indoleacrylic acid	35.38	ND	41.19	ND	64.92	ND
3‐Indolepropionic acid	**14.88**	42.57	**22.84**	91.15	**7.91**	45.97
Indole‐3‐carboxyaldehyde	**12.97**	**20.94**	**17.08**	143.4	**7.58**	**22.96**
Indole‐3‐carboxylic acid	ND	**7.59**	ND	110.21	ND	**7.29**
Indole‐3‐lactic acid	83.69	ND	50.1	ND	**18.72**	ND
Serotonin	**10.64**	52.28	89.23	103.93	37.03	50.51
2‐Hydroxypalmitic acid	**16.61**	69.44	35.58	89.25	61.24	80.8
Inosine	ND	45.97	ND	123.11	ND	74.28
Hypoxanthine	**8.32**	**18.97**	**13.48**	91.42	**7.53**	**16.13**
Nicotinamide	106.43	46.91	50.79	99.81	**17.99**	42.92
Pseudouridine	71.71	ND	**16.04**	ND	102.41	ND
Uridine	81.35	**23.39**	**25.42**	132.18	36.45	**21.41**
3‐Methyl‐2‐oxovalerate	**13.55**	**29.66**	**9.75**	86.42	**16.79**	30.04
Cortisone	54.87	ND	40.46	ND	54.35	ND
No. of metabolites with CV < 30	6	5	6	0	6	4

*Note:* Grouped summary of fecal metabolites detected in this study. Metabolites are grouped by chemical classes, and their relative abundance is expressed as a percentage (%). CV values are shown for each metabolite under the three normalization methods. Metabolites with CV values ≤ 30, highlighted in bold, indicate high technical reproducibility.

Fresh fecal samples exhibited lower variability (CV < 30) than kit‐stored samples, although the latter yielded a greater number of detectable metabolites. In fresh stool, both wet and dry weight normalization effectively reduced variation, whereas in kit samples, wet weight normalization consistently provided the highest reproducibility. By contrast, dry weight normalization of kit samples failed to produce any metabolites meeting the CV < 30 criterion, likely due to inconsistencies introduced during the drying process. Direct analysis of the kit solution without a drying step improved chromatographic clarity and reproducibility (Figure 3), suggesting a practical adjustment for large‐scale studies where workflow efficiency and consistency are critical.

Figure 3Chromatograms comparing the relative abundance based on metabolite type. (a) Amino acids_proline, (b) bile acids_cholic acid, and (c) carnitines_butyrylcarnitine. Red, dry weight normalization; green, wet weight normalization; black, protein normalization.

**Figure:**
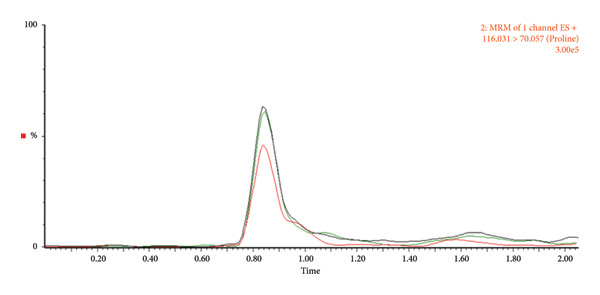
(a)

**Figure:**
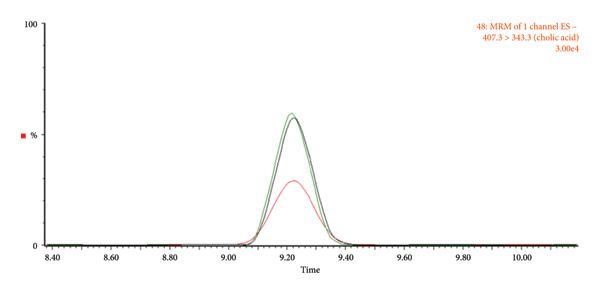
(b)

**Figure:**
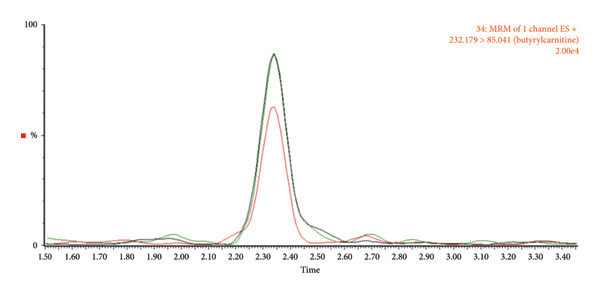
(c)

These results reinforce a core principle in microbial metabolomics: functional metabolic outputs can remain relatively stable despite variability in microbial composition. This finding aligns with previous observations in other complex microbial systems, where consistent functional outputs are maintained even under high taxonomic variability [18]. Similarly, Schott et al. [19] demonstrated that wet weight normalization yields stable bile acid measurements in fresh feces, while Tarazona Carrillo et al. [20] highlighted the importance of standardized homogenization procedures to enhance reproducibility. Collectively, our findings underscore the value of standardized pretreatment and normalization approaches to capture reliable biological signals in fecal metabolomics, particularly for multiomics studies that integrate microbiome and metabolome data.

Feces represent a heterogeneous biological matrix shaped by gut microbiota–host interactions, diet, and digestion [21]. Variability in sample weights, particularly in self‐collected or kit‐based samples, further complicates quantitative analyses [20]. Our results demonstrate that reproducible normalization strategies validated in fresh feces can be applied to OMNIgene·GUT kit samples, supporting their use for simultaneous microbiome and metabolite profiling from the same specimen [22]. This compatibility enables integrative multiomics analyses that can provide deeper insights into microbe–metabolite–host interactions, with applications in clinical, nutritional, and precision medicine research.

Despite these advances, several limitations warrant consideration. The targeted metabolomics approach analyzed only 41 metabolites, which may not fully capture the complexity of the fecal metabolome. Expanding to untargeted approaches would allow broader coverage of gut microbial metabolic activity. In addition, the small number of healthy control samples limited assessment of interindividual variability; larger and more diverse cohorts will be essential for validating reproducibility across populations. Finally, this study evaluated only one type of microbiome preservation kit, and further work is needed to assess the generalizability of these normalization strategies across different kits, storage conditions, and stool consistencies.

## 4. Conclusions

This study provides the first systematic evaluation of fecal metabolite normalization strategies across fresh and OMNIgene·GUT‐preserved stool samples. We demonstrated that wet weight normalization consistently yields the most reproducible metabolite profiles, while direct analysis of the kit solution without drying further improves reproducibility and workflow efficiency.

These findings establish a practical framework for standardizing fecal metabolite analysis and demonstrate the feasibility of using OMNIgene·GUT samples for integrated microbiome–metabolome profiling. This compatibility is particularly valuable for multiomics cohort studies, ensuring reproducible and comparable data across diverse populations and large‐scale research settings.

Looking forward, expanding metabolite coverage with untargeted metabolomics, validating reproducibility across larger and more diverse cohorts, and testing additional preservation strategies will strengthen the applicability of these protocols. By integrating metabolomics with metagenomics, metatranscriptomics, and proteomics, future studies can generate deeper insights into microbe–metabolite–host interactions, ultimately accelerating their applications in precision medicine and personalized nutrition.

## Conflicts of Interest

The authors declare no conflicts of interest.

## Author Contributions

Conceptualization and data curation: Yu Ra Lee and Ho‐Young Park. Formal analysis: Yu Ra Lee. Funding acquisition: Ho‐Young Park. Investigation: Jae‐Ho Park, Hye Hyun Yoo, and Inwook Choi. Methodology: Yu Ra Lee and Hye Hyun Yoo. Resources: Jae‐Ho Park. Supervision: Ho‐Young Park. Writing–original draft: Yu Ra Lee. Writing–review and editing: Jae‐Ho Park, Hye Hyun Yoo, Inwook Choi, and Ho‐Young Park. All authors reviewed the manuscript.

## Funding

This research was supported by the Main Research Program (E0210602) of the Korea Food Research Institute (KFRI) funded by the Ministry of Science and ICT.

## Supporting Information

Table S1. Optimized multiple reaction monitoring condition for simultaneous analysis.

## Supporting information


**Supporting Information** Additional supporting information can be found online in the Supporting Information section.

## Data Availability

Data are available upon request from the authors.
