# Supplementary material for: Standardization of Fecal Metabolomics Using Microbiome Preservation Kits: Implications for Multiomics Integration
Source: Int J Anal Chem. 2025 Dec 17;2025:8551545. doi: 10.1155/ianc/8551545 (PMC12752882; doi:10.1155/ianc/8551545)
Supplement: Supplementary file 1 — Supporting Information Additional supporting information can be found online in the Supporting Information section. [file IANC-2025-8551545-s001.docx]

**Standardization of fecal metabolomics using microbiome preservation kits: Implications for multi-omics integration**

Yu Ra Lee ^1^, Jae-Ho Park ^1^, Hye Hyun Yoo ^2^, Inwook Choi ^1^, Ho-Young Park ^1,3,*^

^1^ *Food Functionality Research Division, Korea Food Research Institute, Jeollabuk-do 55365, Republic of Korea*

^2^ *Pharmacomicrobiomics Research Center, College of Pharmacy, Hanyang University, Ansan 15588, Republic of Korea*

^3^ Department of Food Biotechnology, Korea National University of Science and Technology, Daejeon 34113, Republic of Korea

*** Correspondence:**

Ho-Young Park, Ph.D.

Korea Food Research Institute; 245 Nongsaenmyeong-ro, Wanju-gun, Jeollabuk-do 55365, Republic of Korea

E-mail: [hypark@kfri.re.kr](mailto:hypark@kfri.re.kr); Telephone: +82-63-219-9347; Fax: +82-63-219-9876

**Table S1.** Optimized multiple reaction monitoring condition for simultaneous analysis

| **Group** | **Compound** | **Precursor ion** | **Product ion** | **Collision energy** | **Retention time (min)** |
| --- | --- | --- | --- | --- | --- |
| Amino acids | 4-Hydroxyproline | 132.1 | 86.2 | 14 | 0.9 |
|  | Arginine | 175.1 | 70.2 | 20 | 0.87 |
|  | Aspartate | 134.1 | 74.1 | 14 | 0.93 |
|  | Betaine | 118.1 | 59.1 | 16 | 0.95 |
|  | Cystine | 241.1 | 152 | 12 | 0.88 |
|  | Glutamine | 148.1 | 84.2 | 14 | 0.92 |
|  | Histidine | 156.1 | 110.2 | 14 | 0.86 |
|  | Isoleucine | 132.1 | 86.2 | 10 | 1.43 |
|  | Leucine | 132.1 | 86.2 | 12 | 1.55 |
|  | L-Ornithine | 133.1 | 70.2 | 14 | 0.87 |
|  | Lysine | 147.1 | 84.2 | 14 | 0.84 |
|  | Methionine | 150.1 | 104.1 | 12 | 1.23 |
|  | *N*-alpha-Acetyllysine | 189.3 | 84.1 | 20 | 0.84 |
|  | Phenylalanine | 166.1 | 120.1 | 14 | 2.17 |
|  | Proline | 117 | 71.1 | 14 | 0.94 |
|  | Serine | 106 | 60.2 | 10 | 0.9 |
|  | Threonine | 120 | 74.1 | 8 | 0.92 |
|  | Tryptophan | 205.1 | 146.1 | 18 | 3.16 |
|  | Tyrosine | 182.1 | 136.1 | 12 | 1.29 |
|  | Valine | 118.1 | 72.2 | 10 | 1.25 |
|  | Kynurenine | 209.1 | 146.1 | 18 | 2.23 |
| Bile acids | Cholic acid | 407.3 | 343.3 | 30 | 12.71 |
|  | Deoxycholic acid | 391.3 | 345.2 | 28 | 14.52 |
|  | Taurocholic acid | 514.3 | 80.1 | 58 | 9.89 |
| Carnitines | L-Carnitine | 162.1 | 60.2 | 16 | 0.94 |
|  | Lauroylcarnitine | 344.4 | 85 | 24 | 13.14 |
| Fatty acids | 2-Hydroxypalmitic acid | 271.3 | 225.2 | 20 | 17.83 |
| Indoles | 3-Indoleacrylic acid | 188.1 | 115.1 | 28 | 7.94 |
|  | Indole-3-carboxaldehyde | 146.1 | 118.1 | 14 | 6.33 |
|  | Serotonin | 177.1 | 115.1 | 24 | 1.74 |
| Purines | Hypoxanthine | 137 | 110.1 | 20 | 1.29 |
